# Quantitative Confounder Analysis of Electrocardiogram Signals in Cardiac Magnetic Resonance at 1.5, 3 and 7 T—Assessing Standardized Electrode Positions and Sequence Types—Towards Quality Assurance

**DOI:** 10.1002/jmri.70130

**Published:** 2025-10-04

**Authors:** Richard Hickstein, Stephanie Wiesemann, Darian Viezzer, Denise Kleindienst, Teodora Chitiboi, Bogdan Andrei Gheorghita, Jens Wetzl, Thomas Hadler, Sebastian Dietrich, Sebastian Schmitter, Jeanette Schulz‐Menger

**Affiliations:** ^1^ Working Group Cardiovascular Magnetic Resonance, Experimental and Clinical Research Center, Charité Medical Faculty, Max‐Delbrück Center for Molecular Medicine, Helios Klinikum Berlin Buch, Department of Cardiology and Nephrology Charité—Universitätsmedizin Berlin, Kardiologie—ECRC Berlin Germany; ^2^ DZHK (German Centre for Cardiovascular Research) Berlin Germany; ^3^ Siemens Healthineers AG Forchheim Germany; ^4^ Advanta, Siemens SRL Brasov Romania; ^5^ Physikalisch‐Technische Bundesanstalt (PTB) Berlin Germany

**Keywords:** 7 T, cardiac gating, cardiac gating confounders, ultra‐high field

## Abstract

**Background:**

The electrocardiogram (ECG) used for gating in cardiac MRI may be compromised by multiple confounders inside the scanner bore.

**Purpose:**

To quantify the influence of magnetic field strengths (1.5 T/3 T/7 T), standardized electrode positions, and imaging sequences on ECG signals used for gating.

**Study Type:**

Prospective.

**Population:**

Sixteen healthy volunteers (eight male; mean age 26.25 ± 7.67 years).

**Field Strength/Sequence:**

Balanced steady‐state free precession cine (1.5 T/3 T), fast low‐angle shot cine (7 T), and 4D flow (1.5 T/3 T/7 T) sequences.

**Assessment:**

ECG‐signals were recorded during breath‐hold and non‐breath‐hold short axis cine (sax‐bh and sax‐nbh, respectively) and 4D flow scans at 1.5 T/3 T/7 T. All scans were repeated with 4 standardized electrode positionings (pos1–4) at each field strength. Pos1/2 were vendor‐recommended positionings for 1.5 T/3 T/7 T scans, respectively, whereas pos3/4 were alternative positionings recommended in previous studies. Similarity between confounded ECG‐signals and unconfounded baseline ECG‐signals was assessed by QRS‐feature correlation. Cine image quality (IQ) was assessed by 3 readers (with 6, 10, and 22 years experience) on a four‐point Likert scale.

**Statistical Tests:**

Linear mixed models with type III tests of fixed effects (overall) and *t* tests with adjusted degrees of freedom (pairwise subgroup‐comparisons) at significance level *p* < 0.05.

**Results:**

Increasing field strength resulted in significantly decreasing similarity to baseline measurements, with *r* values (provided with 95% confidence interval) of 1.5 T: 97% (92.6–101.3); 3 T: 91.4% (87.1–95.8); 7 T: 50.4% (46–54.9) and lower IQ: 1.5 T: 2.33 (2.12–2.55); 3 T: 1.96 (1.75–2.17); 7 T: 0.91 (0.7–1.12). Vendor‐specified electrode positions pos1: 91.8% (87.2–96.5), pos2: 88.3% (83.7–92.9) showed significantly higher correlation with baseline measurements than alternative positions pos3: 67.5% (62.9–72.1) and pos4: 70.8% (66.2–75.4). The evaluated standardized sequences showed similar amounts of electrocardiogram distortion, with *r* values of: sax‐bh: 77.3% (73–81.7); 4D: 79.3% (75–83.7), *p* = 0.54; sax‐nbh: 82.1% (77.8–86.5), *p* = 0.31, but the difference between sax‐bh and sax‐nbh: 4.8% (2.88–6.72) was significant.

**Data Conclusion:**

Increasing field strength leads to significant ECG signal distortions. Vendor‐specified positions 1/2 resulted in less distorted ECG signals than alternative positions 3/4 recommended in previous publications.

**Level of Evidence:**

2.

**Technical Efficacy:**

Stage 5.

## Plain Language Summary

The electrical signal of the heart plays an important role in synchronizing cardiac MR imaging with the heart's motion. This study measured how much this signal is distorted by magnetic field strengths (1.5, 3, and 7 T), electrode positioning, and imaging sequences. The results showed higher amounts of distortion with increasing field strengths but less distortion with standard electrode positions. The tested imaging sequences showed little difference. These results highlight challenges for ultra‐high‐field MRI in clinical practice and provide practical guidance to optimize electrode placement. They may pave the way for more reliable heart imaging at increasing magnetic field strengths.

## 1 Introduction

Cardiac MRI is accepted as the reference standard for assessing cardiac function, volumes, and morphology [1]. To compensate for cardiac motion, most MRI techniques rely on synchronizing the image acquisition with the cardiac cycle (cardiac gating). The electrocardiogram (ECG) used for cardiac gating is typically derived from three or four surface electrodes [2] and is not of diagnostic quality [3]. Most commonly, cardiac gating is achieved by detecting the R‐peak in the characteristic QRS complex of the ECG signal [4]. Inside the MR scanner environment, however, the ECG signal is often compromised [3] due to interference from radiofrequency (RF) pulses and switching magnetic field gradients [5]. In addition, moving charged particles (ions) in the blood act as additional confounders as, inside the magnet bore, they experience a Lorentz force. This force is oriented perpendicular to the main magnetic field and is linearly proportional to the field strength and the ions' velocity [6, 7]. The deflected ions generate an electric potential across the vessel, especially in the aortic arch, where the blood flow direction is perpendicular to the main field. This potential superimposes the ECG signal and is strongest during the QT segment due to the blood flow in the aorta in this cardiac phase, at approximately 107 ms after the R‐wave [6]. Since this magnetohydrodynamic (MHD) effect increases with field strength, the quality of the ECG signal, and thus the gating performance, decreases from 1.5 to 7 T [7, 8, 9]. To overcome the MHD effect on the ECG signal, there have been attempts to develop new trigger detection methods [10, 11]: A vectorcardiogram (VCG) approach has been developed to improve R‐peak detection accuracy [4] and is currently the standard method for clinical field strengths of 1.5 and 3 T. At 7 T, however, there have been mixed findings regarding the performance of VCG‐based gating [12, 13].

Several studies have emphasized the role of electrode positioning for good ECG signal quality at clinical field strengths [3, 14]. Furthermore, some studies have recommended alternative electrode positions for use in cases where the vendor‐recommended standard positioning does not provide a sufficiently reliable signal [9, 15, 16, 17]. Although there have been attempts to explore alternative electrode positions at 7 T [17], the impact of electrode positioning on ECG signal quality and thus gating performance at 7 T remains to be investigated. In a clinical setting, finding an electrode positioning providing sufficient signal quality is usually performed iteratively [3], starting from vendor‐recommended standard positions and moving to alternative positions, and sometimes with image‐guided optimization [18]. Although this process often leads to successful results, it may be time‐consuming especially for ultra‐high field (UHF, > = 7 T) strengths [6]. Consequently, investigating the impact of standardized electrode positions on ECG signal quality at UHF strengths may be an important step on the way to bringing ultra‐high field cardiac MRI into clinical practice.

Besides magnetic field strength and electrode positioning, a previous study has also highlighted the confounding impact of imaging sequences on ECG gating signals, suggesting a decrease in signal quality for sequences using strong gradients and rapid switching [19]. Thus, various filtering methods have been developed to compensate for these effects [20, 21]. With rapid switching of very strong gradients, however, the ECG signal may still be affected [19, 22].

**FIGURE 1:**
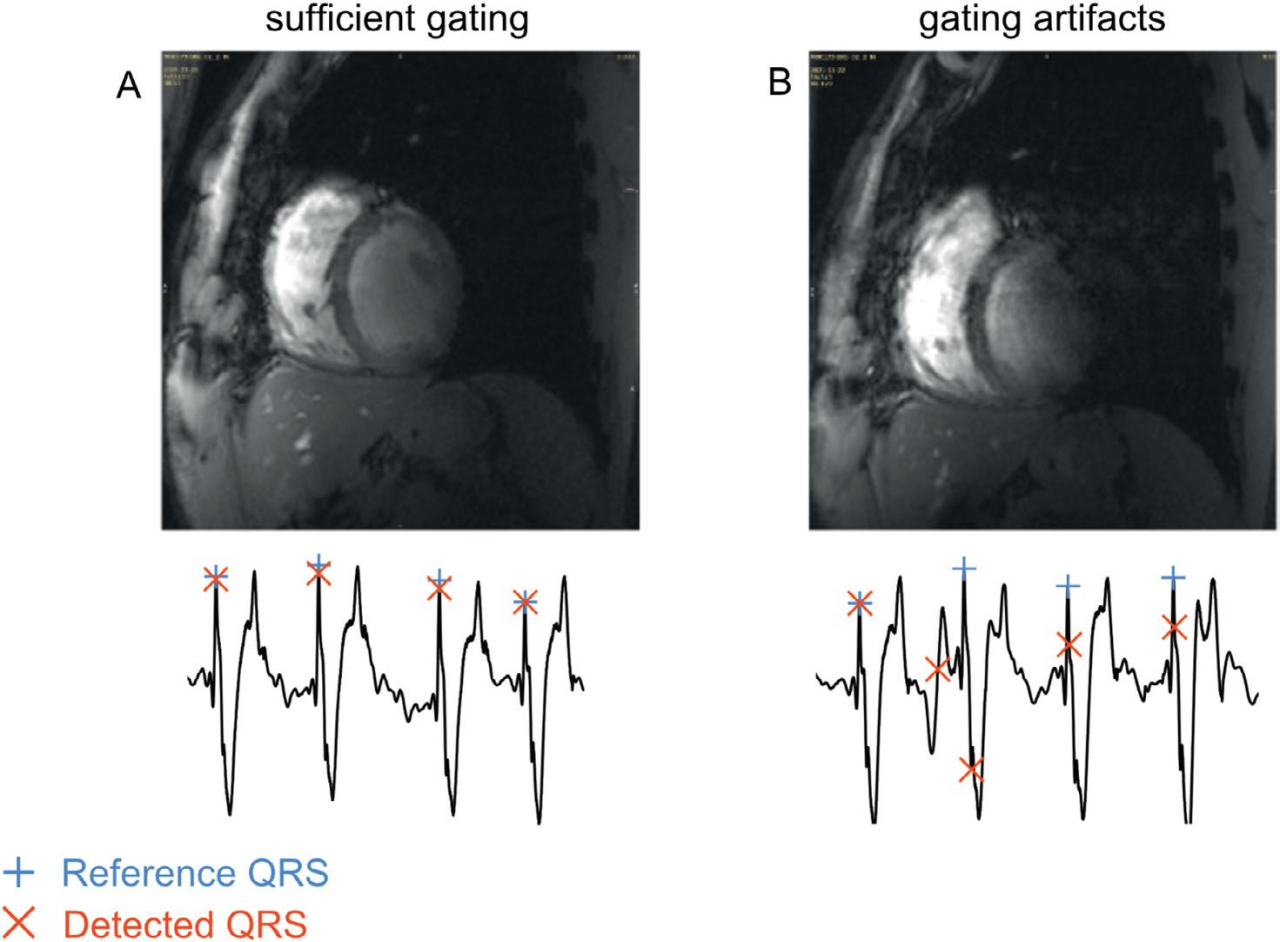
Gating artifacts. Mid‐ventricular short axis breath‐hold slices acquired at 7 T. Good quality images (A) can be obtained when the ECG signal quality is sufficient for cardiac gating. However, ECG signal distortion may result in impaired gating performance, which can lead to ghosting artifacts and thus, a loss of image quality (B). Example ECG signals and QRS‐triggers, as detected by the vendor's default algorithm, are marked in red. Manually defined reference QRS‐triggers are marked in blue. ECG, electrocardiogram.

The confounding influence of the MHD effect, electrode position, and gradient switching accumulates. Therefore, severe gating problems may occur, especially at UHF strengths, leading to increased scan durations, errors in quantification of clinical parameters due to image artifacts (Figure 1), and sometimes even failed measurements [23]. The importance of these confounders remains only partially understood, especially at UHF strengths. Counteracting their impact, however, requires a quantifiable measure to assess each individual confounder's effect on ECG signal quality. Furthermore, a quantitative comparison could also aid in estimating dropout rates and scan durations for MRI studies, allowing for pre‐scan optimization of MR protocols and setups.

Thus, the aim of this study was to quantify and compare the influence of four standardized electrode positions and three standardized MRI sequences on ECG signal quality at clinical field strengths of 1.5, 3, and at 7 T.

## 2 Materials and Methods

### 2.1 Population

This study was approved by the local ethics board (EA1/183/19). All volunteers provided written informed consent before enrollment. Sixteen volunteers (8 male, 8 female) aged 19–52 years (mean: 26.25 ± 7.67 years), with body mass index (BMI) ranging from 18.6 to 29.73 kg/m^2^ (mean: 23.94 ± 3.15 kg/m^2^) and without contraindications for MRI or known cardiovascular disease were included.

### 2.2 Field Strengths

To quantify the impact of magnetic field strength on ECG signals, all volunteers were scanned at 1.5, 3, and 7 T (MAGNETOM Avanto fit, MAGNETOM Skyra fit, and MAGNETOM 7 T, respectively, Siemens Healthineers AG, Forchheim, Germany).

### 2.3 Electrode Positions

**FIGURE 2:**
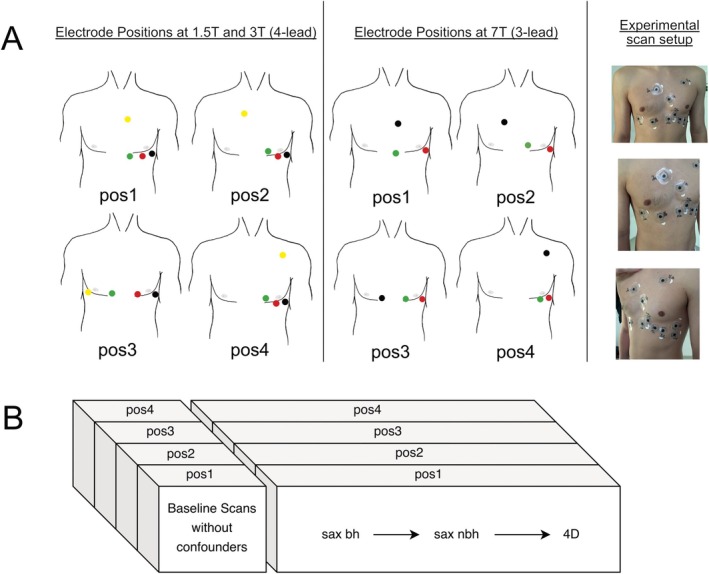
Standardized electrode positions and protocol overview. (A) Standardized electrode placement positions for clinical (left) and ultra‐high field strength (middle) studies. The far‐right image shows the four electrode positions on a volunteer's chest. Note that some of the electrodes were reused for multiple positions. The electrode colors in the schematic drawings are as in the vendor manuals [24, 25]. (B) An overview of the protocol: For all four electrode positions at each field strength, baseline (unconfounded) ECG signals were acquired with the volunteer outside the scanner while running a Localizer sequence gated by this baseline signal. This procedure was necessary for later extraction of the ECG signal from the raw data. These were followed by ECG signal measurements during short‐axis cine scans with breath‐hold and non‐breath‐hold, and 4D flow scans. 4D, 4D flow; bh, breath‐hold; nbh, non breath‐hold; pos, standardized electrode position; sax, short axis.

To assess the impact of electrode positioning on the ECG signal, four standardized electrode positions (pos) were defined as follows (Figure 2A).

1. Position 1 (pos1): based on vendor recommendations for a 3‐lead ECG setup as used in the 7 T scanner [24].
2. Position 2 (pos2): based on vendor recommendations for clinical field strengths with a 4‐lead setup [25] as used in the 1.5 and 3 T scanners.
3. Position 3 (pos3): based on recommendations for alternative electrode positions at ultra‐high field MRI [6, 9].
4. Position 4 (pos4): an adaptation of recommended alternative ECG‐lead positions on the back [26] and the antero‐lateral thorax [17].

Both pos1 and pos2 are described in the systems' operator manuals [24, 25] and are consistent with studies recommending precordial leads to reduce MHD‐related artifacts [15, 16]. Pos3/4 differ notably from the vendor‐recommended positions (pos1/2) and were selected in this study to investigate a wide range of electrode positioning schemes. To compensate for the differing number of electrodes available at the scanners in this study, the additional fourth electrode available at 1.5 and 3 T was placed at the midway point between the other two electrodes used in the same position at 7 T (Figure 2A).

To realize all 4 positions, a total of 11 (1.5 and 3 T) or 9 (7 T) electrodes were attached to the volunteer's skin at the beginning of the session by an MR technician with 22 years of MRI experience (DK) according to a standardized illustrated manual (Figure 2A) and following general recommendations on patient preparation and electrode application, including shaving the volunteer's chest prior to placing the electrodes when necessary [3].

### 2.4 MRI Protocol and Sequences

First, the ECG signal was recorded during baseline scans for all four electrode positions (pos1–4) to later serve as a reference for the confounded ECG signals obtained inside the scanner. At 1.5 and 3 T, balanced steady‐state free precession (bSSFP) and at 7 T, fast low angle shot (FLASH) based localizers served as baseline scans. To minimize any confounding impact of the magnetic field on the ECG signal during these baseline measurements, the volunteer was placed outside the scanner bore, with a water‐based phantom placed in the magnet's isocenter. Imaging a phantom was necessary for later extraction of the volunteer's ECG signal from the raw data (refer to the next section). At 7 T, the volunteer was placed approximately 2 m away from the bore on a separate patient table. At 1.5 and 3 T, the wireless receiver's range restricted the maximum distance from the patient table to the scanner bore to approximately 1.5 m. For each of the four electrode positions, ECG signals were then recorded with the volunteer inside the scanner while running short‐axis view (sax) cine and 4D‐flow acquisitions (4D), with 4D‐flow chosen to represent a sequence type with high switching gradients [27] (refer to Figure 2B). Each scan was performed twice using the system's default ECG and VCG gating algorithms. For the sax scans, bSSFP (1.5 T, 3 T) and FLASH gradient echo (FLASH‐GRE) sequences (7 T) were used, as is common in clinical cardiac MRI at these field strengths [28]. Sax data were acquired with and without breath holding (sax bh and sax nbh, respectively) [28]. Sequence parameters (Table 1) at 1.5 and 3 T were typical of those used in clinical studies in our hospital. The 7 T protocol was newly set up, aiming to closely match the parameters of the 3 T protocol. Each individual measurement's scan duration was adjusted to collect an ECG signal of > 90 s duration per scan, except for breath‐hold acquisitions, which were set to acquire 12 k‐space segments/beat, resulting in ~15 beats/acquisition due to physiological constraints.

**TABLE 1:** Scan parameters.

Field strength	1.5 T			3 T			7 T		
Sequence	bSSFP localizer	bSSFP sax bh/nbh	4D	bSSFP localizer	bSSFP sax bh/nbh	4D	FLASH localizer	FLASH sax bh/nbh	4D
FOV	340 × 276 mm^2^ (81.3%)	340 × 276 mm^2^ (81.3%)	360 × 293 mm^2^ (81.3%)	400 × 400 mm^2^ (100%)	360 × 302 mm^2^ (84%)	400 × 325 mm^2^ (62.5%)	250 × 250 mm^2^ (100%)	360 × 259 mm^2^ (71.9%)	400 × 325 mm^2^ (81.3%)
Matrix size	192 × 156	192 × 156	160 × 88 × 4	256 × 168	208 × 122	192 × 120 × 6	256 × 225	256 × 184	192 × 156 × 4
Slice thickness	6 mm	6 mm	3.6 mm	8 mm	6 mm	2.1 mm	7 mm	4 mm	2.1 mm
Resolution	1.8 × 1.8 × 6 mm^3^	1.8 × 1.8 × 6 mm^3^	2.3 × 2.3 × 3.6 mm^3^	1.6 × 2.4 × 8 mm^3^	1.7 × 2.5 × 6 mm^3^	2.1 × 2.1 × 2.1 mm^3^	1.0 × 1.1 × 7 mm^3^	1.4 × 1.4 × 4 mm^3^	2.1 × 2.1 × 2.1 mm^3^
TR	33.36 ms	33.36 ms	42.4 ms	478.09 ms	50.52 ms	187.2 ms	8.6 ms	71.04 ms	170.4
TE	1.19 ms	1.19 ms	2.9 ms	1.13 ms	1.84 ms	4.7 ms	4 ms	3.57 ms	4.2
Nominal flip‐angle	50°	74°	8°	51°	52°	15°	20°	12°	15°
TA planned	> 90 s	12 segments/beat (bh) > 90 s (nbh)	> 90 s	> 90 s	12 segments/beat (bh) > 90 s (nbh)	> 90 s	> 90 s	12 segments/beat (bh) > 90 s (nbh)	> 90 s
Venc (in‐plane)			1.5 m/s			1.5 m/s			1.5 m/s
Venc (through‐plane)			1.5 m/s			1.5 m/s			1.5 m/s

*Note*: All sequences used a Cartesian trajectory. bSSFP/FLASH Localizer scans were used as baseline scans for the QRS‐feature correlation (refer to methods). Note that the parameters for the sax bh and sax nbh sequences are the same except for TA: the TA for the nbh scans was set to acquire > 90 s of confounded ECG signal samples during the sequence. The bh scans could not be set to 90 s due to physiological constraints. Therefore, they were set to acquire 12 k‐space segments/beat resulting in ~15 beats/scan. Note that for 4D flow, the duration was set to record a minimum of 90 s of confounded ECG signal samples but not necessarily longer to reduce overall scan time.

Abbreviations: bh, breath‐hold; bSSFP, balanced steady‐state free precession; FOV, field of view; nbh, non breath‐hold; sax, short axis; TA, acquisition time; TE, echo time; TR, repetition time.

### 2.5 ECG Signal Extraction and Post‐Processing

ECG signals were recorded at a sampling rate of 400 Hz for all scans. At 1.5 and 3 T, the ECG signal was extracted from the raw MR data via prototype software provided by the vendor and truncated to the actual scan duration using time stamps from the raw MR data.

**FIGURE 3:**
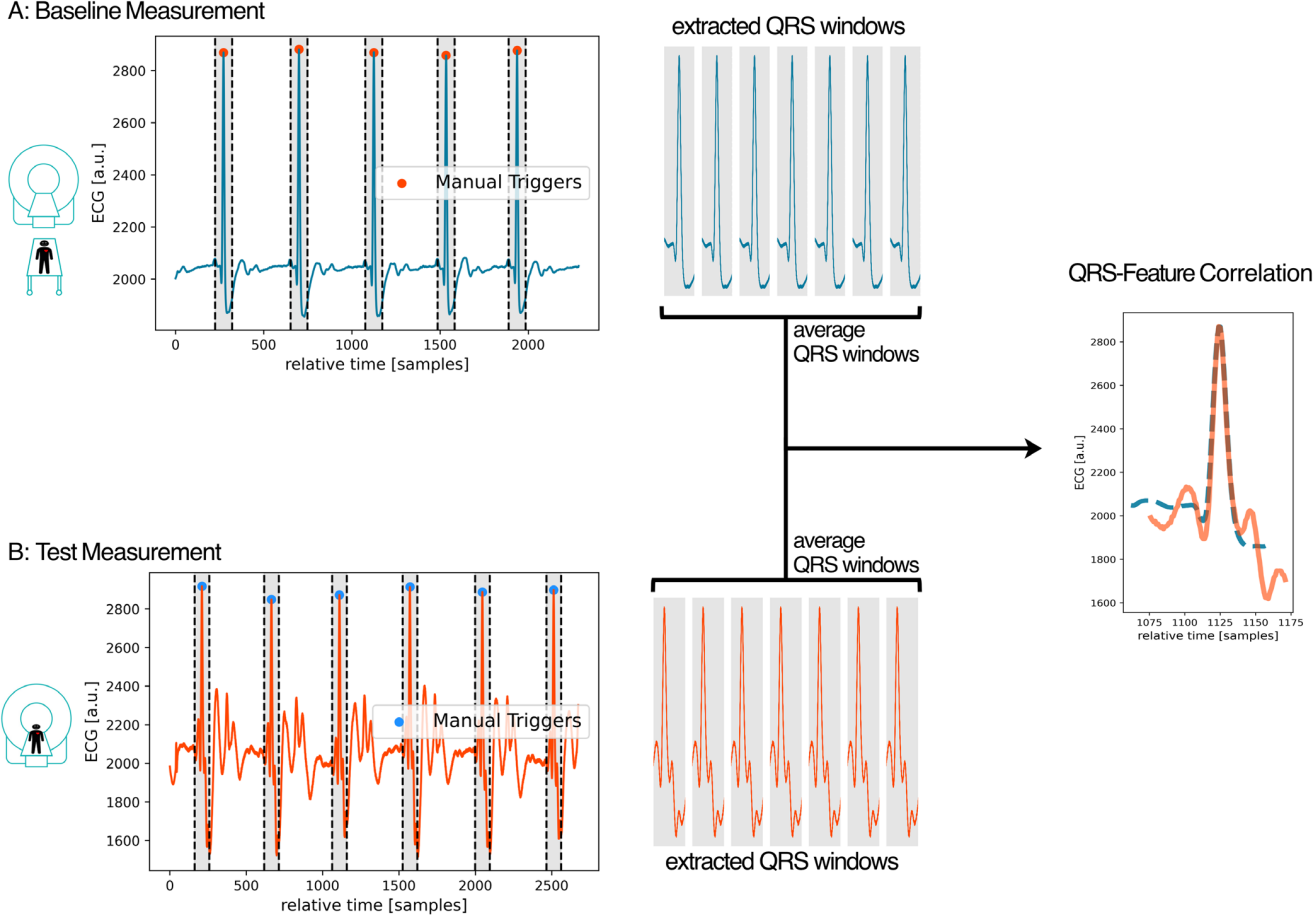
QRS‐feature‐correlation. (A) Unconfounded baseline ECG signal measurement (volunteer outside the scanner). (B) Test measurement (volunteer inside the scanner, ECG signal confounded by field strength (1.5, 3, and 7 T), electrode position (pos1–4), and sequence (short‐axis cine with/without breath‐hold, 4D flow sequence)). QRS‐features were extracted by averaging the signal vectors of all 120 ms windows around manually detected R‐peaks to calculate a QRS feature vector. The QRS feature for the test measurement was then compared to the corresponding baseline measurement's QRS feature (same field strength, same electrode position, and same gating method) using Pearson's correlation coefficient (*r* values) as a similarity measure to quantify the amount of distortion introduced by the confounders in the test measurement. a.u., arbitrary unit; ECG, electrocardiogram.

At 7 T, no such tool was available; hence, the ECG signal was recorded manually for each scan via physiologging from the scanner console. The data was transferred to a web‐based prototype annotation tool (Siemens Medical Solutions, Princeton, NJ, USA) for manual R‐peak trigger annotation by a reader with 6 years of clinical ECG signal analysis (RH). A QRS‐feature vector was extracted from each measurement by averaging the vectors of ECG signal samples of all 120 ms windows around manually determined R‐peaks [29] for comparison of confounded test and unconfounded baseline measurements (refer to statistical analysis and Figure 3).

### 2.6 Subjective Image Quality Analysis

Image quality of the cine images was analyzed using a four‐point Likert scale (0 = poor due to gating artifacts, non‐diagnostic; 1 = fair, diagnostics may be impaired; 2 = good, some gating artifacts but no interference with diagnostics; 3 = excellent, no gating artifacts) as described previously [30] by 3 independent readers with 6 (RH), 22 (DK), and 10 years of MRI experience.

### 2.7 Statistical Analysis

To quantify the impact of field strength, electrode position, and imaging sequence type on the quality of the ECG signal, the QRS features of confounded ECG signals were compared with their corresponding (unconfounded) baseline QRS feature, that is the feature generated from the ECG signal measurement with the same electrode position, gating method, and field strength but with the volunteer outside the scanner. Similarity between confounded and baseline QRS features was assessed by calculating Pearson's correlation coefficient (*r*) between the two extracted feature vectors according to:

$$r_{\mathrm{xy}}=\frac{\sum_{i=1}^{n}\left(x_{i}-\bar{x}\right)\left(y_{i}-\bar{y}\right)}{\sqrt{\sum_{i=1}^{n}{\left(x_{i}-\bar{x}\right)}^{2}}\sqrt{\sum_{i=1}^{n}{\left(y_{i}-\bar{y}\right)}^{2}}}$$

where $r_{\mathrm{xy}}$ denotes Pearson's correlation coefficient between x and y, where x is the QRS‐feature vector extracted from the confounded test measurement (i.e., the averaged 120 ms window around manually denoted R‐peaks) and y is the QRS‐feature vector extracted similarly from the corresponding baseline measurement.

Post‐processing was implemented in the programming language Python 3.11.3 using the NumPy 1.24.3 [31] and pandas 2.0.3 [32] libraries.

To compensate for repeated measurements within individual volunteers, linear mixed models were applied, treating the volunteers as a random effect and the confounders field strength (1.5, 3, and 7 T), sequence type (4D and sax bh/nbh), and electrode position (pos1‐4) as fixed effects [33]. To adjust for a differing number of observations per confounder—for example in case a measurement for a combination such as 7 T‐pos3‐4D failed in a subset of volunteers—least squares means (LS means), that is adjusted means derived from the model instead of standard averages, were used. Each confounder's (field strength, sequence type, position) impact on the ECG signal (*r*‐values) and image quality was tested for using type III tests of fixed effects while adjusting for the remaining confounders and their interactions. A stepwise hierarchical test strategy was used: If significance was shown in the global test (e.g., testing if field strength has an effect on ECG signals), significance was assessed in pairwise comparisons between measurements in the subgroups using *t*‐tests with degrees of freedom adjusted for potentially imbalanced group sizes [34]. Differences between subgroups defined by bivariable combinations (e.g., 1.5 T‐pos2 vs. 3 T‐pos4) and trivariable combinations (e.g., 1.5 T‐pos2‐4D vs. 3 T‐pos4‐sax‐nbh) of the assessed confounders were tested. A *p* value < 0.05 was considered significant. The statistical analyses were performed in the dedicated statistical programming language R (version 4.3.2) [35] using the lmerTest 3.1.3 (for statistical testing) [33] and lsmeans 2.30.0 (for LS means difference calculations) [34] packages.

## 3 Results

### 3.1 Data Acquisition

Out of 1536 ECG measurements acquired, 1447 could be included for analysis (94.2%). Of the 89 measurements (5.8%) which had to be excluded, 9 (0.6%) were excluded due to ECG‐file corruption, 34 (2.2%) due to ECG signal extraction failure, 42 (2.7%) due to data transfer problems, and in 4 cases (0.2%), the scan failed completely due to insufficient gating. The total number of manually detected R‐peaks in the included measurements was 111,932.

### 3.2 Impact of Field Strength

**FIGURE 4:**
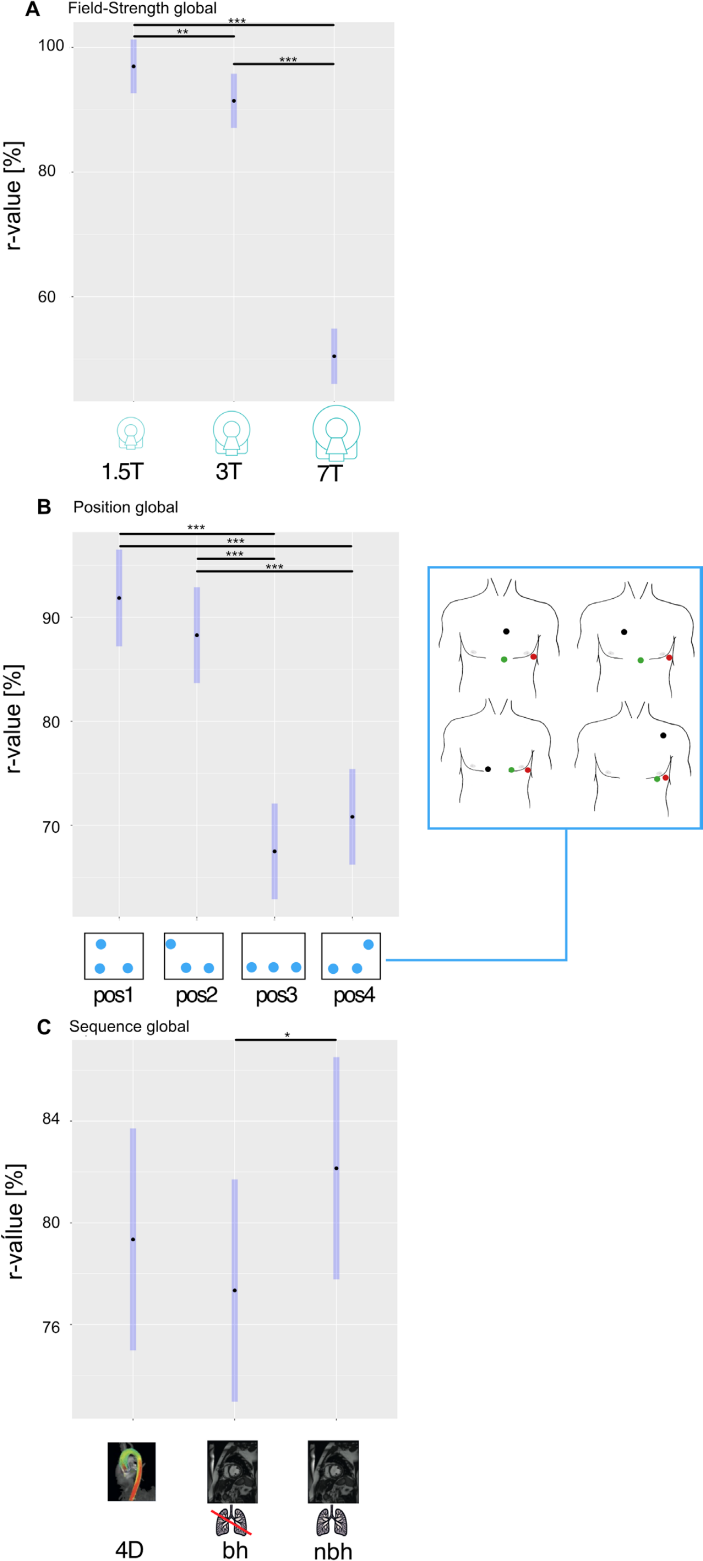
Global results: Impact of field strength, electrode position, and sequence on ECG signals. Least‐square mean *r* values for (A) field strengths of 1.5, 3, and 7 T. (B) Electrode positions 1–4 (pos1–4), and (C) 4D flow and short‐axis cine sequences with and without breath‐hold across all measurements (global). Blue bars indicate 95% confidence intervals. Significant differences are shown with a bar and an asterisk (*p < 0.05, **p < 0.01, ***p < 0.001). 4D, 4D flow; bh, breath‐hold; ECG, electrocardiogram; nbh, non breath‐hold; pos, standardized electrode position.

Increasing field strength (1.5, 3, and 7 T) led to a significant reduction in ECG signal similarity to baseline signals. All values are provided with 95% confidence intervals (CI). LS mean *r* values of 1.5 T: 97% (92.6–101.3) versus 3 T: 91.4% (87.1–95.8) versus 7 T: 50.4% (46–54.9) were found (Figure 4A). Differences between subgroups are provided in the supplement. Subjective image quality of the sax cine acquisitions also varied significantly with field strength, with mean scores of 1.5 T: 2.3 (2.12–2.55) versus 3 T: 1.96 (1.75–2.17) versus 7 T: 0.91 (0.7–1.12).

### 3.3 Impact of Electrode Position

**FIGURE 5:**
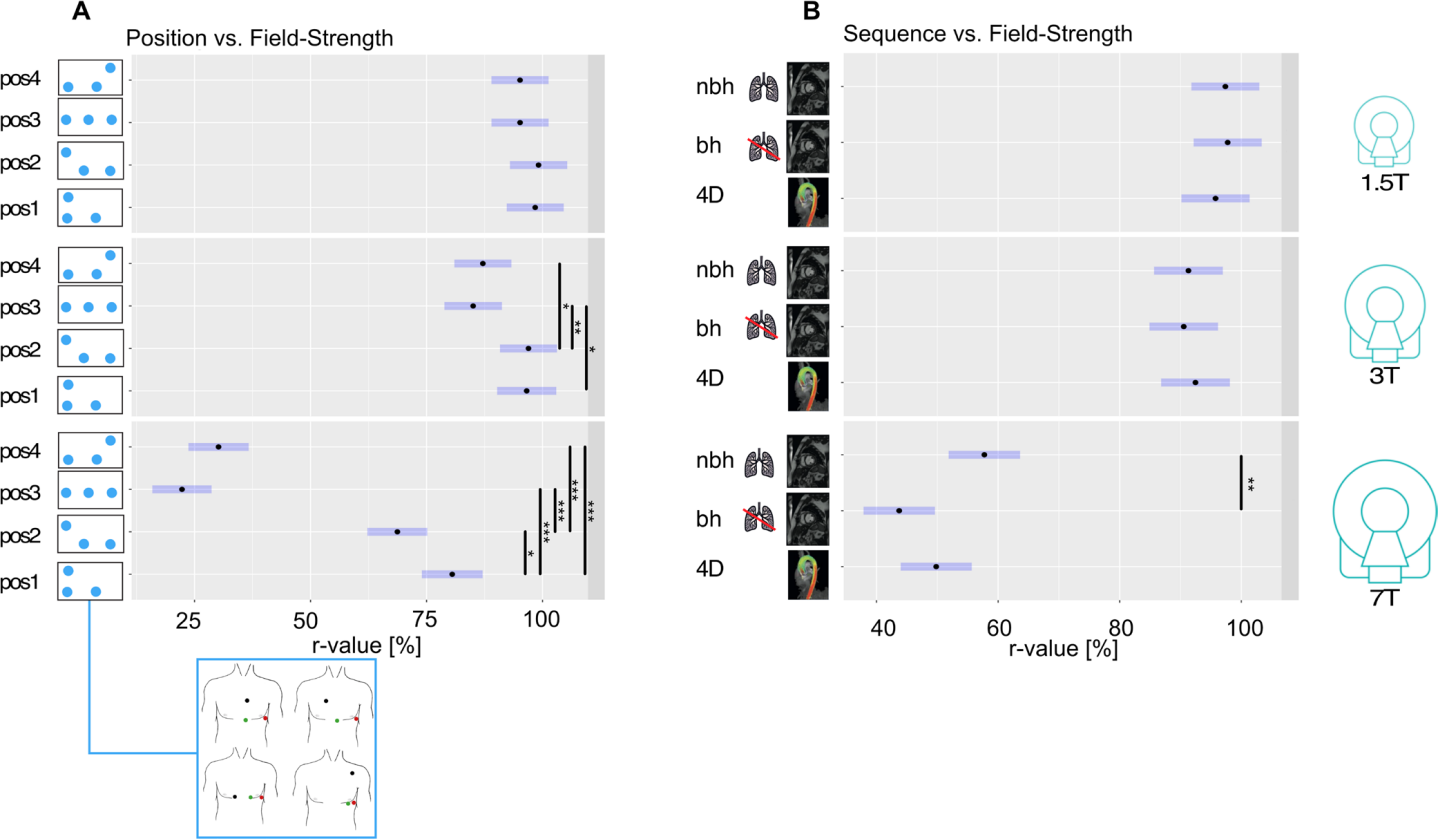
Subgroup analysis for combinations of field strength/position and field strength/sequence. Least‐square mean *r* values for (A) positions (pos1‐4), and (B) 4D flow and short‐axis cine sequences with and without breath‐hold at 1.5 T (top), 3 T (middle), and 7 T (bottom). Blue bars indicate 95% confidence intervals. Significant differences are shown with a bar and asterisk (*p < 0.05, **p < 0.01, ***p < 0.001).4D, 4D flow; bh, breath‐hold; nbh, non breath‐hold; pos, standardized electrode position.

**FIGURE 6:**
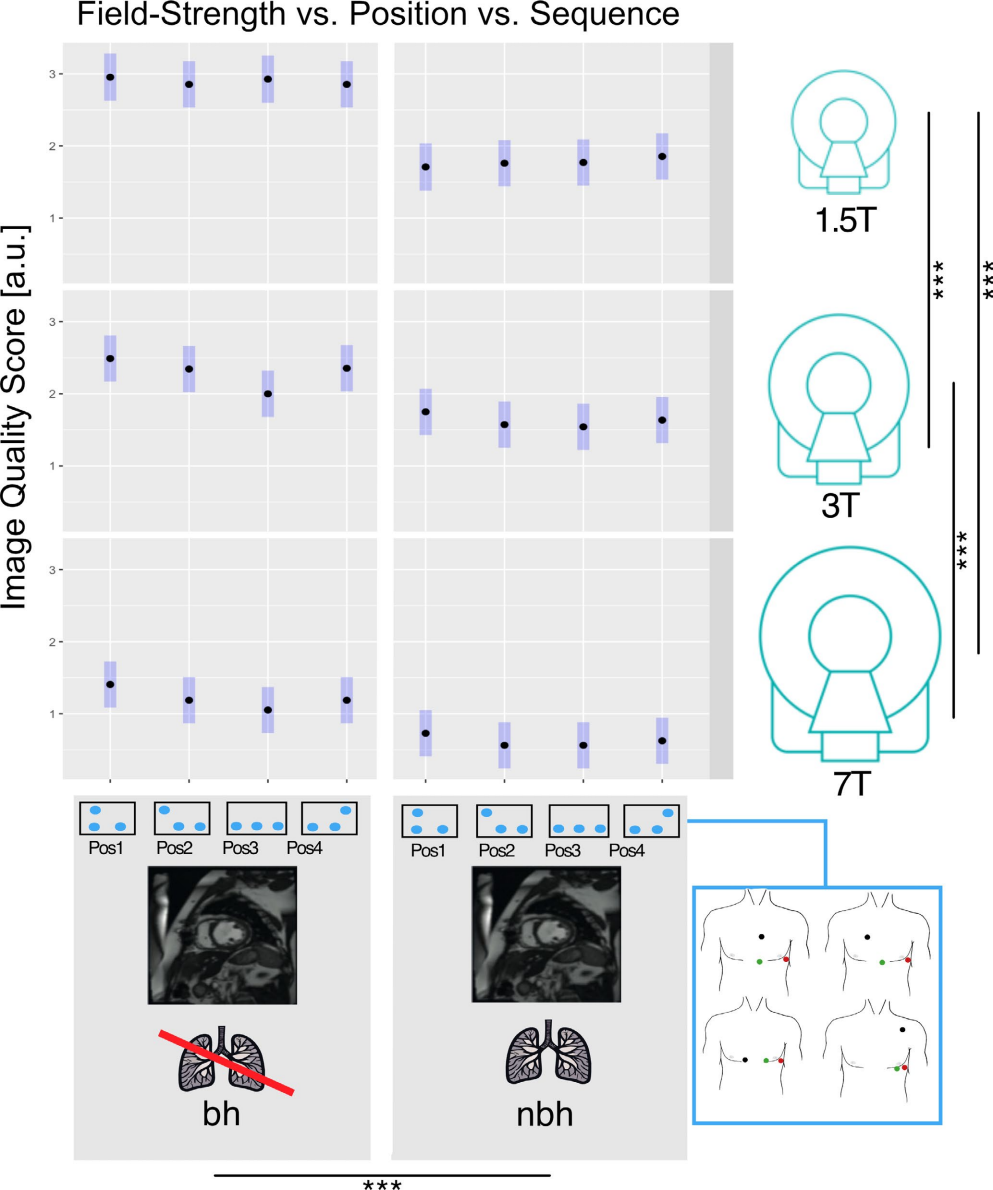
Image quality analysis for field strength, position, and sequence. Average image quality scores based on a four‐point Likert scale (0 = poor/non‐diagnostic; 1 = fair, 2 = good; 3 = excellent) as rated by three blinded observers for short axis cine images acquired with and without breath‐hold using standardized electrode positions 1–4 at 1.5 T (top), 3 T (middle), and 7 T (bottom). Blue bars indicate 95% confidence intervals. Significant differences are shown with a bar and asterisk (*p < 0.05, **p < 0.01, ***p < 0.001). bh, breath‐hold; nbh, non breath‐hold; pos, standardized electrode position.

The four evaluated electrode positions showed significantly different *r* values when compared across all field strengths and sequences, with pos1 providing the highest correlation with baseline measurements and thus the best signal quality (Figure 4B). Overall, vendor‐recommended positions (pos1/2) and alternative positions (pos3/4) showed no significant differences within the same group (*r* value differences of pos1 vs. pos2: 3.57% (1.34–5.8), *p* = 0.38 and pos3 vs. pos4: 3.32% (1.12–5.52), *p* = 0.43), but there was a significant difference between vendor‐recommended positions (pos1/2) versus alternative positions (pos3/4) (Table 2). In the subgroup analysis, comparing the four standardized positions at individual field strengths (1.5, 3, or 7 T), vendor‐recommended positions (pos1/2) showed no significant difference to alternative positions (pos3/4) at 1.5 T (all *p* > 0.70). At 3 and 7 T, however, pos1/2 showed significantly less ECG signal distortion than alternative positions (pos3/4), except for pos4‐pos1 at 3 T (*p* = 0.06), with a larger difference between pos1/2 and pos3/4 at 7 T (Figure 5A). The subjective image quality scores of the cine acquisitions (provided as LS mean and 95% CI) were pos1: 1.84 (1.62–2.06) versus pos2: 1.71 (1.50–1.93) versus pos3: 1.64 (1.43–1.86) versus pos4: 1.75 (1.54–1.97), with no significant difference when compared across all measurements (all *p* > 0.05). There were no significant differences in image quality scores for the standardized electrode positions at individual field strengths (all *p* > 0.3) except for pos3‐pos1 at 3 T (Figure 6).

**TABLE 2:** Global comparison.

Global comparison		*r*‐value difference estimate [%]	SE [%]	*p*
Field strength	3 T–1.5 T	−5.53	1.89	**0.009**
	7 T–1.5 T	−46.54	1.96	**< 0.001**
	7 T–3 T	−41.01	1.97	**< 0.001**
Position	Pos2–pos1	−3.57	2.23	0.379
	Pos3–pos1	−24.35	2.23	**< 0.001**
	Pos3–pos2	−20.78	2.20	**< 0.001**
	Pos4–pos1	−21.03	2.23	**< 0.001**
	Pos4–pos2	−17.45	2.21	**< 0.001**
	Pos4–pos3	3.32	2.20	0.432
Sequence	Sax bh–4D	−2.01	1.93	0.548
	Sax nbh–4D	2.81	1.93	0.312
	Sax nbh–sax bh	4.81	1.93	**0.033**

*Note*: Three different field strengths (1.5, 3, and 7 T). Four standardized electrode positions. Standardized sequences: 4D flow, short‐axis cine sequence with and without breath‐hold. The comparisons were performed across all measurements (global) marginalizing over the other confounders for example 3–1.5 T compared the adjusted mean *r*‐values of all measurements at 3 T with the adjusted mean *r*‐values of all measurements at 1.5 T regardless of electrode position or sequence. Significance was assumed at *p* < 0.05. Significant differences marked in bold.

Abbreviations: 4D, 4D flow; bh, breath‐hold; nbh, non breath‐hold; pos, standardized electrode position; sax, short axis; SE, standard error.

### 3.4 Impact of Sequence Type

Overall, there was no significant difference between the effect of the 4D flow sequence and the short‐axis cine sequences (4D‐sax bh: −2.01% (−3.93, −0.09), *p* = 0.55; sax nbh‐4D: 2.81% (0.88–4.72), *p* = 0.31). A small 4.8% (2.88–6.72), but significant difference was found between sax nbh and sax bh (Figure 4C). In the subgroup analysis, comparing the standardized sequences at individual field strengths, this effect was more pronounced at 7 T: 14.01% (10.51–17.48), with no significant differences between sax nbh and sax bh at clinical field strengths: 1.5 T: −0.37% (−3.60–2.86), *p* = 0.99 and 3 T: 0.77% (−2.50–4.04), *p* = 0.97 (Figure 5B). The subjective image quality analysis showed significant differences between sax bh: 2.13 (1.93–2.34) and sax nbh acquisitions: 1.34 (1.13–1.54).

### 3.5 Combinations of Field Strength, Electrode Position, and Sequence

**FIGURE 7:**
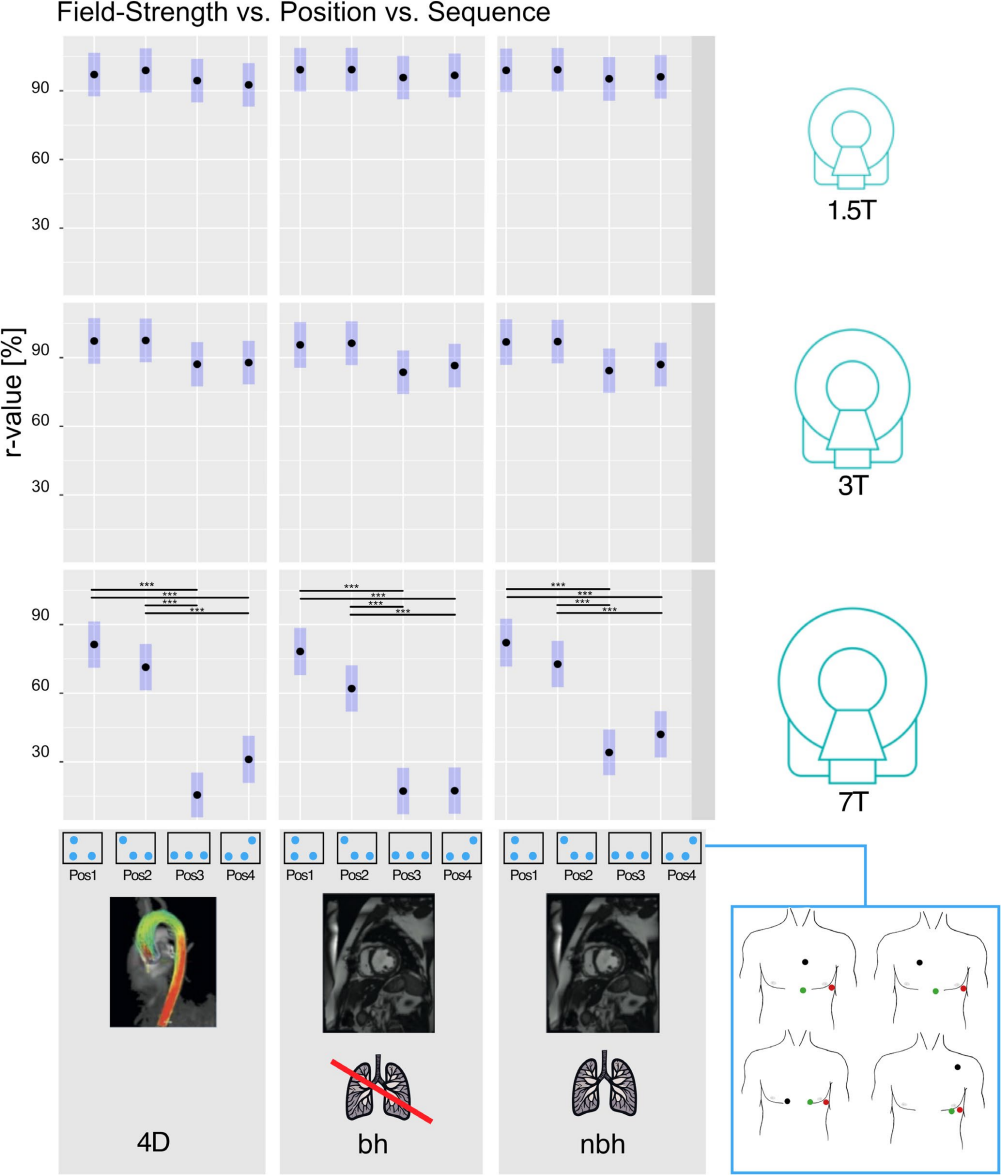
Subgroup analysis for combinations of field strength, position, and sequence. Least‐square mean *r* values for combinations of position (pos1–4) and sequence (4D flow, short‐axis cine with and without breath‐hold) at 1.5 T (top), 3 T (middle), and 7 T (bottom). Blue bars indicate 95% confidence intervals. Significant differences are shown with a bar and asterisk (*p < 0.05, **p < 0.01, ***p < 0.001). 4D, 4D flow; bh, breath‐hold; nbh, non breath‐hold; pos, standardized electrode position.

LS mean *r* values for all combinations of field strength, electrode position, and sequence are presented in Figure 7 showing that increasing field strength results in lower *r* values. The largest differences in *r* values between electrode positions were also observed at 7 T.

## 4 Discussion

This study quantified changes in ECG signals recorded at field strengths of 1.5, 3, and 7 T, with four standardized electrode positions during standardized 4D flow and sax cine (bh/nbh) acquisitions.

### 4.1 Impact of Field Strength

In line with previous studies, increased ECG signal distortions were observed with increasing field strength, which led to a decrease in image quality. This may be explained by an increase in the MHD effect with increasing field strength [36]. In this study, the confounding effect on the ECG signal was quantified by a similarity measure (the QRS‐feature correlation coefficient) at 1.5, 3, and 7 T under scanning conditions resembling clinical study setups, that is during image acquisition using the gating hardware of the scanner. This approach builds on previous studies that have targeted the MHD at clinical or ultra‐high field strengths but were limited to non‐MRI‐conditional ECG equipment that did not allow quantifying the confounding effect of different sequence types [37].

Our findings may facilitate estimating gating problems in the planning stage of MRI studies and could potentially assist in estimating the potential number of scan failures or anticipating prolonged scan durations.

ECG signal distortions may lead to a complete loss of trigger detection and thus failed image acquisition. In this study, the number of scans where a complete loss of trigger detection occurred was consistent with a previous study [38].

To overcome the problem of field strength dependent artifacts on the ECG signal, gating techniques such as pulse wave triggering [38] or the acoustic cardiogram (ACT) [9] have been suggested as alternatives for ECG signal‐based gating, especially for ultra‐high‐field strengths. While pulse wave triggering is limited by its latency to the R‐peak, the ACT detects the first heart tone of the phonocardiogram, the acoustic signal produced by the contraction of the heart during systole [39]. It is not affected by the magnetic field strength, making it attractive for UHF studies. However, the ACT uses additional hardware and has limitations due to variable time delays between the R‐peak and the first heart tone [4]. Furthermore, variable success rates have been reported [40, 41].

Another innovative technique derives cardiac gating signals from a modulation of the pilot tone, a continuous RF‐signal outside of the frequency range of the MR‐imaging [40], and several studies have shown promising results at clinical field strengths [40, 42]. However, the associated commercially available device (Biomatrix Beatsensor, Siemens Healthineers AG, Forchheim) is not yet available for 7 T systems, although a similar approach has been discussed [43]. Recently, machine‐learning‐based approaches have also been proposed for gating in cardiac MRI [44]. Furthermore, self‐gating, which compensates for cardiac motion directly based on the image data using dedicated post‐processing, has been proposed [45]. However, while these innovative approaches have shown promising results, they require additional hard‐ or software. Furthermore, due to the wide availability and ease of use of the ECG in clinical practice, methods relying on the electrical signal of the heart remain the current clinical standard for gating [28]. However, with the development of modern gating techniques, this might change in the future. On the other hand, the ongoing development of low‐field MRI techniques, which are less affected by the MHD [46], may result in continued application of ECG‐signal‐based gating techniques.

### 4.2 Impact of Electrode Position

Recent studies have demonstrated that a reduction of ECG signal distortions may be possible when including additional ECG channels, for example using a 12‐channel ECG device and custom trigger detection algorithms [37, 47]. However, the 12‐channel ECG recorders in these studies were used without pulse gradient switching and in a research environment only, with only limited experience in a clinical setting [47]. Until ECG devices with more channels become widely available for gated image acquisition, optimizing the electrode placement of the available channels remains of high importance [3, 15].

In this study, the suggested alternative positions (pos3/4) provided similar ECG signals and image quality to the standard positions (pos1/2) recommended by the vendor at 1.5 T. At 1.5 T, our results therefore support the use of standardized alternative positions as a starting point for a streamlined research‐based approach for electrode setup optimization in cases where the vendor‐recommended positions do not provide sufficient gating performance.

At 3 T/7 T, vendor recommended pos1/2 resulted in significantly less ECG signal distortion compared to the alternative positions from previous studies (pos3 and pos4), although the image quality obtained with the latter was not significantly reduced, also rendering them potential candidates for a streamlined electrode position optimization approach. These findings may be explained by variations in the thorax configurations of the patients, which can also influence ECG signals obtained from surface electrodes [14]. This confounding effect may have had a stronger impact on the ECG signals in our study than in the previously published studies discussed above [6, 9, 17, 26], possibly leading to higher amounts of distortion with the alternative electrode positions in this study. A direct quantitative comparison to previous studies, however, is not feasible due to different study designs and setups [17, 48]. To provide further insight on the confounding role of different thorax configurations on ECG gating signals, please refer to the Supporting Information.

### 4.3 Impact of Sequence Type

Our results showed no significant differences in ECG signal similarity between sax cine and 4D sequences at 1.5, 3, or 7 T. This contrasts with previous studies reporting increasing distortions of the ECG signal due to rapid gradient switching from pulse sequences used for image acquisition [19, 21, 22]. These findings may be explained by the improved filtering methods developed in recent years [19, 21, 49] and the use of high impedance cables attenuating induced currents from the imaging gradients [50]. In addition, differences in the sequences and their parameters may have led to different results.

Furthermore, significantly higher ECG distortion was observed for sax bh acquisitions than sax nbh acquisitions, mainly at 7 T. This may be explained by the fact that the duration of the sax bh measurements was set shorter (~15 s) than the sax nbh measurements due to physiological constraints. Hence, an inability to hold breath during the breath‐hold maneuver may have had a higher impact on feature correlation than repeated shallow breathing, since the technique averages multiple ECG signal windows across time in the calculation of the QRS feature. The longer duration of the sax nbh measurements would also explain the lower subjective image quality scores observed for sax nbh measurements, with image quality being affected by respiratory motion in addition to cardiac gating, but does not explain why higher signal distortions were observed mainly at 7 T. In general, a standardized procedure for quantifying ECG signal distortions in MRI environments would be beneficial for assessing confounders and comparing innovative gating methods. As the characteristic R peak is the most stable feature of typical ECG curves in a healthy population [10], it can be assumed that the shape of the ECG signal's R peak stays constant across a scan's duration without any external confounders such as field strength or arrhythmic disorders influencing the ECG signal itself. This assumption holds, even for ECG signals in a cardiac MRI environment [3]. Therefore, in this study, a feature correlation approach was used to quantify the effect of external confounders on ECG signal quality. In QRS feature correlation, ECG signal distortions are quantified by comparison of confounded QRS features with a corresponding baseline feature, enabling the quantification of each individual confounders' impact on the signal. Thus, the method could also be applied to investigate other confounders not assessed in this study.

### 4.4 Limitations

Due to the nature of this pilot study, the number of volunteers was relatively small (*n* = 16). Therefore, the statistical analyses were adjusted for repeated measurements within individual volunteers to obtain as much information on the assessed confounders as possible within the frame of an exploratory study. Furthermore, since the healthy volunteers had no history of cardiovascular disease, the results may not be fully applicable to patients in a clinical setting.

Subjective image quality was limited to sax cine sequences, since scan duration restrictions required temporal adjustments of the 4D measurements.

## 5 Conclusion

Increasing field strength has a significant negative impact on ECG signal quality. At 1.5 T, vendor‐recommended electrode positions and standardized alternative positions may be used interchangeably, whereas at 3 T/7 T the vendor‐recommended positions (pos1/2) resulted in less ECG distortion than alternative positions (pos3/4), yet provided comparable image quality. Sax cine and 4D sequences showed no significant difference in ECG signal distortion at 1.5 and 3 T in this study, although a small but significant difference between bh and nbh cine acquisitions was observed at 7 T.

## Conflicts of Interest

S.S. received funding from Deutsche Forschungsgesellschaft SCHM 2677/21 and GRK2260. J.W., T.C., and B.A.G. are employees and/or affiliates at Siemens Healthineers AG.

Carsten Schwenke provides consultation services to Experimental and Clinical Research Center on an honorary basis.

## Supporting information


**Table S1:** Least squares mean difference estimates for bivariate combinations between field‐strength (1.5, 3, and 7 T) and standardized sequences (4D flow, short‐axis cine sequence with breath‐hold and without breath‐hold).
**Table S2:** Least squares mean difference estimates for bivariate combinations between field‐strength (1.5, 3, and 7 T) and standardized electrode positions.
**Figure S1:** Thorax configuration as a confounder.

**Data S1:** jmri70130‐sup‐0002‐DataS1.pdf.

## Data Availability

The raw data cannot be made available due to federal data privacy and protection rules.
