# Supplementary material for: Quantitative Confounder Analysis of Electrocardiogram Signals in Cardiac Magnetic Resonance at 1.5, 3 and 7 T—Assessing Standardized Electrode Positions and Sequence Types—Towards Quality Assurance
Source: J Magn Reson Imaging. 2025 Oct 4;63(4):1140–54. doi: 10.1002/jmri.70130 (PMC12963813; doi:10.1002/jmri.70130)
Supplement: Supplementary file 1 — Table S1: Least squares mean difference estimates for bivariate combinations between field‐strength (1.5, 3, and 7 T) and standardized sequences (4D flow, short‐axis cine sequence with breath‐hold and without breath‐hold). Table S2: Least squares mean difference estimates for bivariate combinations between field‐strength (1.5, 3, and 7 T) and standardized electrode positions. Figure S1: Thorax configuration as a confounder. [file JMRI-63-1140-s002.docx]

| Field-strength/sequence  comparison | | estimate [%] | se [%] | p-value |
| --- | --- | --- | --- | --- |
| 1.5T | sax bh – 4D | 2.00 | 3.23 | 0.811 |
|  | sax nbh - 4D | 1.63 | 3.23 | 0.871 |
|  | sax nbh - sax bh | -0.38 | 3.23 | 0.993 |
| 3T | sax bh - 4D | -1.92 | 3.27 | 0.827 |
|  | sax nbh - 4D | -1.15 | 3.28 | 0.935 |
|  | sax nbh - sax bh | 0.78 | 3.27 | 0.970 |
| 7T | sax bh - 4D | -6.09 | 3.45 | 0.182 |
|  | sax nbh - 4D | 7.93 | 3.45 | 0.057 |
|  | sax nbh - sax bh | 14.01 | 3.47 | **<0.001** |

Supplementary Table 1: Least squares mean difference estimates for bivariate combinations between field-strength (1.5T, 3T, 7T) and standardized sequences (4D flow, short-axis cine sequence with breath-hold and without breath-hold

**se:** standard error; **4D:** 4D flow; **sax:** short axis; **bh:** breath hold; **nbh:** non breath-hold

| Field strength/position  comparison | | estimate [%] | se [%] | p-value |
| --- | --- | --- | --- | --- |
| 1.5T | pos2 - pos1 | 0.72 | 3.74 | 0.997 |
|  | pos3 - pos1 | -3.26 | 3.73 | 0.817 |
|  | pos3 - pos2 | -3.99 | 3.74 | 0.710 |
|  | pos4 - pos1 | -3.27 | 3.73 | 0.816 |
|  | pos4 - pos2 | -4.00 | 3.74 | 0.708 |
|  | pos4 - pos3 | -0.01 | 3.73 | 1. |
| 3T | pos2 - pos1 | 0.39 | 3.83 | 1. |
|  | pos3 - pos1 | -11.50 | 3.84 | **0.015** |
|  | pos3 - pos2 | -11.90 | 3.75 | **0.008** |
|  | pos4 - pos1 | -9.44 | 3.83 | 0.066 |
|  | pos4 - pos2 | -9.82 | 3.73 | **0.042** |
|  | pos4 - pos3 | 2.10 | 3.75 | 0.944 |
| 7T | pos2 - pos1 | -11.80 | 4.02 | **0.018** |
|  | pos3 - pos1 | -58.20 | 3.99 | **<.001** |
|  | pos3 - pos2 | -46.40 | 3.95 | **<.001** |
|  | pos4 - pos1 | -50.40 | 4.04 | **<.001** |
|  | pos4 - pos2 | -38.50 | 4.00 | **<.001** |

Supplementary Table 2: Least squares mean difference estimates for bivariate combinations between field-strength (1.5T, 3T, 7T) and standardized electrode positions

**se:** standard error; **pos:** standardized electrode position


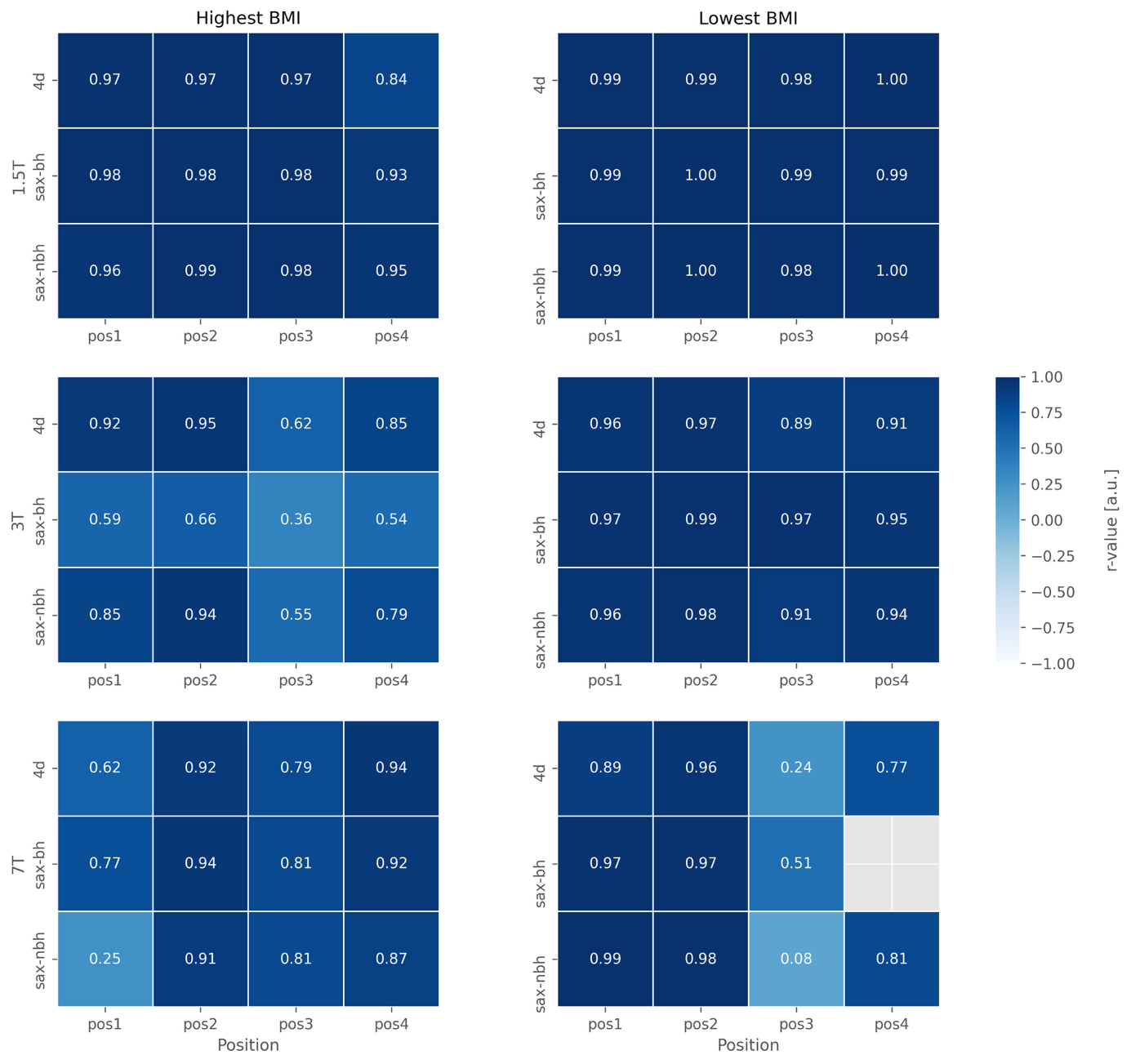


Supplementary Figure 1: Thorax configuration as a confounder

The Figure displays the r-values for the volunteer with the highest BMI (29.72 kg/m^2^) and the lowest BMI (18.59 kg/m^2^) for field strengths of 1.5T, 3T and 7T, standardized electrode positions 1-4 and standardized sequences (4D flow, short-axis cine sequence with breath-hold and without breath-hold). For 7T-pos4-sax-bh, gating failed, therefore the entry is left blank. Interestingly, the volunteer with the lowest BMI showed higher ECG-signal-distortions than the volunteer with the highest BMI especially at 7T. This may be explained by the narrower intercostal space in patients with a lower BMI.

**pos:** standardized electrode position, **4D:** 4D flow; **sax:** short axis; **bh:** breath hold; **nbh:** non breath-hold
