# Supplementary material for: Quantitative Confounder Analysis of Electrocardiogram Signals in Cardiac Magnetic Resonance at 1.5, 3 and 7 T—Assessing Standardized Electrode Positions and Sequence Types—Towards Quality Assurance
Source: J Magn Reson Imaging. 2025 Oct 4;63(4):1140–54. doi: 10.1002/jmri.70130 (PMC12963813; doi:10.1002/jmri.70130)
Supplement: Supplementary file 2 — Data S1: jmri70130‐sup‐0002‐DataS1.pdf. [file JMRI-63-1140-s001.pdf]

1 QRS Feature correlation

For an ECG-Signal measurement σ we take the vector of signal values

$$\mathbf{w}_i = \sigma[t_i - L : t_i + L - 1]$$

with $\frac{L}{2}$ corresponding to a window size of $\pm$ 60ms around each manually annotated R-peak (120ms window) in the ECG signal. We then calculate the average of this window to obtain our QRS feature vector for the measurement (same electrode position, but with the volunteer outside the scanner):

$$\mathbf{x} = \frac{1}{n} \sum_{i=1}^{n} w_i$$

For each confounded QRS feature vector we then calculate pearson's correlation coefficient to quantify how much the test measurement is confounded with regard to its corresponding baseline measurement:

$$\rho_{\mathbf{xy}} = \frac{\sum_{i=1}^{n} (x_i - \bar{x})(y_i - \bar{y})}{\sqrt{\sum_{i=1}^{n} (x_i - \bar{x})^2} \sqrt{\sum_{i=1}^{n} (y_i - \bar{y})^2}}$$

where:

- $\rho_{\mathbf{xy}}$ denotes the pearson correlation coefficient between $\mathbf{x}$ and $\mathbf{y}$
- $\mathbf{x}$ is the confounded test measurement QRS-feature vector
- $\mathbf{y}$ is the baseline measurement QRS-feature vector
- $\bar{x} = \frac{1}{n} \sum_{i=1}^{n} x_i$ denotes the mean of $\mathbf{x}$
- $\bar{y} = \frac{1}{n} \sum_{i=1}^{n} y_i$ denotes the mean of $\mathbf{y}$

2 Statistical Analysis

Consider our linear mixed model:

$$\mathbf{y} = \mathbf{X}\boldsymbol{\beta} + \mathbf{Z}\mathbf{b} + \boldsymbol{\epsilon},$$

where:

- $\mathbf{y}$ denotes the vector of observed responses (our r values),
- $\mathbf{X}$ denotes the fixed-effects design matrix,
- $\boldsymbol{\beta}$ denotes the vector of fixed-effect coefficients,
- $\mathbf{Z}$ denotes the random-effects design matrix,
- $\mathbf{b} \sim N(\mathbf{0}, \mathbf{G})$ are random effects,

- $\boldsymbol{\epsilon} \sim N(\mathbf{0}, \mathbf{R})$ denotes the residual error.

Note that $\mathbf{G}$ and $\mathbf{R}$ are estimated via maximum likelihood (a method for estimating parameters of statistical distributions) during model fitting. The *least squares mean* (or *estimated marginal mean*) for a given combination of fixed-effect factors (in our case ECG signal confounders field strength, electrode position and sequence type) is:

$$\hat{\mu}_i = \mathbf{c}_i^\top \hat{\boldsymbol{\beta}},$$

where:

- $\hat{\boldsymbol{\beta}}$ denotes the best linear unbiased estimator (BLUE) of the fixed effects,
- $\mathbf{c}_i$ denotes a contrast vector corresponding to the factor (confounder) level combination.

The *variance* of the *least squares* mean can then be calculated as:

$$\mathrm{Var}(\hat{\mu}_i) = \mathbf{c}_i^\top \, \mathrm{Var}(\hat{\boldsymbol{\beta}}) \mathbf{c}_i,$$

where

$$\mathrm{Var}(\hat{\boldsymbol{\beta}}) = (\mathbf{X}^\top \mathbf{V}^{-1} \mathbf{X})^{-1}, \quad \mathbf{V} = \mathbf{Z}\mathbf{G}\mathbf{Z}^\top + \mathbf{R}.$$

The *standard error* then is:

$$\mathrm{SE}(\hat{\mu}_i) = \sqrt{\mathrm{Var}(\hat{\mu}_i)}.$$

A 95% *confidence interval CI* can then be constructed by:

$$\hat{\mu}_i \pm t_{\alpha/2,df} \cdot \mathrm{SE}(\hat{\mu}_i),$$

where $t_{\alpha/2,df}$ is from a t-distribution with approximated degrees of freedom (using Kenward–Roger approximation [1]) and $\alpha = 0.05$ in our case.

For two combinations (e.g. 1.5T-pos3-4D and 7T-pos4-sax-bh) denoted by i and j, the variance of their difference is

$$\mathrm{Var}(\hat{\mu}_i - \hat{\mu}_j) = (\mathbf{c}_i - \mathbf{c}_j)^\top \, \mathrm{Var}(\hat{\boldsymbol{\beta}}) (\mathbf{c}_i - \mathbf{c}_j),$$

and thus

$$\mathrm{SE}(\hat{\mu}_i - \hat{\mu}_j) = \sqrt{\mathrm{Var}(\hat{\mu}_i - \hat{\mu}_j)}.$$

The t-test for the difference then uses the statistic

$$t = \frac{\hat{\mu}_i - \hat{\mu}_j}{\mathrm{SE}(\hat{\mu}_i - \hat{\mu}_j)},$$

with degrees of freedom of the t-distribution (for calculating the p-value), again approximated via the Kenward Roger method [1]. The 95% confidence interval for the difference can be computed by

$$(\hat{\mu}_i - \hat{\mu}_j) \pm t_{\alpha/2,df}\ \mathrm{SE}(\hat{\mu}_i - \hat{\mu}_j),$$

with $\alpha = 0.05$.

References

[1] Michael G. Kenward and John H. Roger. “Small sample inference for fixed effects from restricted maximum likelihood”. In: *Biometrics* 53.3 (1997), pp. 983–997. DOI: 10.2307/2533558.
